# Simulation of the cost-effectiveness of malaria vaccines

**DOI:** 10.1186/1475-2875-8-127

**Published:** 2009-06-08

**Authors:** Fabrizio Tediosi, Nicolas Maire, Melissa Penny, Alain Studer, Thomas A Smith

**Affiliations:** 1 Department of Public Health & Epidemiology, Swiss Tropical Institute, Socinstrasse 57, Postfach CH-4002, Basel, Switzerland; 2 Università Bocconi, Milan, Italy

## Abstract

**Background:**

A wide range of possible malaria vaccines is being considered and there is a need to identify which vaccines should be prioritized for clinical development. An important element of the information needed for this prioritization is a prediction of the cost-effectiveness of potential vaccines in the transmission settings in which they are likely to be deployed. This analysis needs to consider a range of delivery modalities to ensure that clinical development plans can be aligned with the most appropriate deployment strategies.

**Methods:**

The simulations are based on a previously published individual-based stochastic model for the natural history and epidemiology of *Plasmodium falciparum *malaria. Three different vaccine types: pre-erythrocytic vaccines (PEV), blood stage vaccines (BSV), mosquito-stage transmission-blocking vaccines (MSTBV), and combinations of these, are considered each delivered via a range of delivery modalities (Expanded Programme of Immunization – EPI-, EPI with booster, and mass vaccination combined with EPI). The cost-effectiveness ratios presented are calculated for four health outcomes, for assumed vaccine prices of US$ 2 or US$ 10 per dose, projected over a 10-year period.

**Results:**

The simulations suggest that PEV will be more cost-effective in low transmission settings, while BSV at higher transmission settings. Combinations of BSV and PEV are more efficient than PEV, especially in moderate to high transmission settings, while compared to BSV they are more cost-effective in moderate to low transmission settings. Combinations of MSTBV and PEV or PEV and BSV improve the effectiveness and the cost-effectiveness compared to PEV and BSV alone only when applied with EPI and mass vaccinations. Adding booster doses to the EPI is unlikely to be a cost-effective alternative to delivering vaccines via the EPI for any vaccine, while mass vaccination improves effectiveness, especially in low transmission settings, and is often a more efficient alternative to the EPI. However, the costs of increasing the coverage of mass vaccination over 50% often exceed the benefits.

**Conclusion:**

The simulations indicate malaria vaccines might be efficient malaria control interventions, and that both transmission setting and vaccine delivery modality are important to their cost-effectiveness. Alternative vaccine delivery modalities to the EPI may be more efficient than the EPI. Mass vaccination is predicted to provide substantial health benefits at low additional costs, although achieving high coverage rates can lead to substantial incremental costs.

## Background

*Plasmodium falciparum *malaria represents one of the world's major causes of morbidity and mortality[1,2] and there is a pressing need for new effective interventions, which, combined with the existing strategies, could effectively reduce the burden of malaria in endemic areas[3].

Among these potential new interventions are vaccines and, although there is currently no licensed malaria vaccine, a number of candidates are under development. The complexity of the malaria life cycle means that a number of different stages of the parasite can be targeted. The candidate that is most advanced in clinical development[4,5] targets pre-erythrocytic stages of the parasite.

Appraisals of candidate malaria vaccines should not include only efficacy and effectiveness evaluations but also cost effectiveness analyses (CEA) aimed at guiding vaccine developers, funding agencies[6], and policy makers to allocate resources so that social and economic benefits are maximized[7,8]. CEA can help in evaluating alternative health interventions because health decision makers are primarily interested in knowing what health improvements can be bought with a given budget, and not the overall economic impact *per se*[9,10]. Previously, the likely epidemiological effects[11] and cost-effectiveness[12] of pre-erythrocytic vaccines when delivered in areas of stable endemic malaria via the Expanded Programme on Immunization (EPI) were estimated based on a dynamic model of malaria epidemiology[13]. These simulations showed that at moderate vaccine prices the cost-effectiveness of such vaccines may be similar to that of other preventive and curative interventions against malaria. However, more evidence is needed on the likely cost-effectiveness of different malaria vaccines under development, and on the implications for it of adopting alternative means of deployment. The cost-effectiveness of a malaria vaccine will depend not only on the vaccine profile and the transmission setting, but also on the vaccination coverage that can be achieved, on the vaccine delivery costs, and of the operational feasibility of the delivery modalities adopted to deploy it.

A companion article to the present one[14], reports on the simulations of the likely epidemiological effects of three different malaria vaccine types: pre-erythrocytic vaccines (PEV), blood stage vaccines (BSV), mosquito-stage transmission-blocking vaccines (MSTBV), and combinations of these. A range of different delivery modalities (EPI, EPI with booster, and mass vaccination combined with EPI) were considered. In this article, both the health system and vaccine delivery costs are attached to the events recorded in these simulations to calculate cost-effectiveness ratios for each deployment strategy and each vaccine, and for each of four health outcomes over a 10-year time-horizon.

## Methods

### Perspective and boundary

The simulations refer to CEA under the perspective of the society as a whole, although only direct costs are included. They thus consider all relevant direct resource inputs and costs to the interventions, and resource consequences, costs, and health impacts resulting from the interventions. The indirect economic impact of malaria such as potential earning forgone of patients and unpaid carers is not included, as its inclusion is controversial in CEA[15,16]. The CEA follows standard cost-effectiveness methodology[8,17-21].

### Interventions being compared

The simulations of vaccines are based on a previously described model for the natural history and epidemiology of *P. falciparum *malaria[13]. This model uses an underlying model based on descriptions of the course of parasite densities in malaria therapy patients[22]. The parameterization of the model for the present simulations is described in the companion article[14] and reviewed in more detail elsewhere[23]. Briefly, each simulated vaccine is characterized by an average initial efficacy (the efficacy reached after completion of a schedule of three doses), and by a half-life of this efficacy, which is assumed to decay exponentially with time. The vaccinated population is assumed to be heterogeneous in the response to vaccination, and to allow for this we assign initial values for efficacy drawn from a beta-distribution[11] (simulated vaccines are delivered at specified ages, and a range of coverage values is considered for vaccination to allow for individuals who do not complete the full schedule).

The effects of the three different vaccine types and four combinations were modeled as follows (see also [14]):

#### (i) Pre-erythrocytic vaccines (PEV)

Pre-erythrocytic vaccines are assumed to lead to a reduction in the proportion of inoculations from the bites of infected mosquitoes that lead to blood stage infection and the vaccine efficacy is assumed to be equal to the proportion by which this force of infection is reduced.

#### (ii) Blood stage vaccines (BSV)

A blood stage vaccine is assumed to reduce parasite densities at each time step by a proportion equal to the vaccine efficacy.

#### (iii) Mosquito stage transmission blocking vaccines (TBV)

Vaccine efficacy is equivalent to the proportional reduction of the probability that a mosquito becomes infected from any one feed on an infectious vaccinated human.

#### (iv) Combination vaccines

Combination vaccines of PEV with TBV, BSV with TBV, BSV with PEV and also a three-way combination of PEV with both BSV and MSTBV are considered. For each combination, PEV and BSV are assumed to be matched in both the initial efficacy and in their rate of decay. Only combinations of PEV, BSV and of PEV-BSV with high efficacy MSTBV are considered since it is unlikely that a MSTBV with low efficacy would be developed.

### Vaccine delivery modalities

The delivery of the three vaccine types and their combinations through the following three strategies are simulated:

#### (i) EPI

Delivery of the vaccines through the EPI according to the usual diphtheria tetanus pertussis (DTP3) vaccine schedule: age 1, 2 and 3 months.

#### (ii) EPI with booster

In addition to the above EPI schedule, this modality includes booster doses at 1, 2, 3 and 4 years after the last EPI schedule. The effect of a booster dose is to restore the protective efficacy to the level achieved after the third dose in the same individual.

#### (iii) Mass vaccination combined with EPI

Delivery via EPI to infants is supplemented with a mass vaccination campaign at the beginning of the intervention period and additional campaigns after five years.

### Vaccine coverage

For vaccines delivered via EPI, the assumed coverage of full vaccination (three doses) corresponds to that reported in Tanzania for three doses of diphtheria tetanus pertussis-hepatitis B virus vaccine in the year 2003, which stood at 89%. The assumed dropout rate from the first to the third dose is 6% since coverage for the first dose of DTP-HBV vaccine was 95%. When booster doses are included, it is assumed that 99% of those that receive the third EPI dose will be given a booster dose 1, 2, 3 and 4 years after the last EPI dose. For mass vaccination the coverage levels of 30, 50 and 70% are simulated.

### Case management model

As detailed in the companion article[14], the simulations of the effects of vaccine interventions use a case management model including both formal and informal treatment, similar to a previous study of the authors[24]. An artemisinin-based combination therapy (ACT), artemether-lumefantrine, is used as first line treatment for uncomplicated malaria, as per recent policy changes, and the drug action model was modified accordingly, both in terms of the potential to reduce rates of severe disease, sequelae and death, and the impact on transmission intensity. The model assumes that 90% of patients comply with the ACT treatment schedule and have a cure rate of 85%, while in non-compliers there is no effect.

### Measurement of health impact

The effect of vaccines is evaluated by simulating the malaria dynamics in a population of 100,000 people over a 10-year time horizon. For each of the seven vaccine options, and each delivery modality, the simulations start from a reference set of assumptions (Table 1 of the companion article[14]). Each of the 21 vaccine schemes is compared with the reference situation at six different transmission intensities to obtain cost effectiveness results for each of the 126 vaccine scenarios in preventing the following outcomes: uncomplicated episodes, severe episodes, deaths and DALYs.

**Table 1:** Vaccine delivery costs – routine EPI – US$ 2006

**Item**	**Source**	**Costs (US$)**		
**Net vacc. purchase cost per dose**	Derived	**1.23**	**2.45**	**12.25**

Vaccine price per dose		1	2	10

Freight	UNICEF estimates	0.0417	0.0417	0.0417

Wastage	WHO estimates	15%	15%	15%

**Distribution per dose**	[29]	**0.09**	**0.09**	**0.09**

**Storage per dose**	[29]	**0.03**	**0.03**	**0.03**

**Management per dose**	[29]	**0.003**	**0.003**	**0.003**

**Delivery per dose**	Derived	**0.13**	**0.13**	**0.13**

*Syringes*		0.06	0.06	0.06

Unit cost per dose	[30]	0.05	0.05	0.05

Freight	UNICEF estimates	0.0417	0.0417	0.0417

Wastage	WHO estimates	10%	10%	10%

*Safety boxes*		0.01	0.01	0.01

Unit cost per dose	[30]	0.0122	0.0122	0.0122

Freight	UNICEF estimates	0.0417	0.0417	0.0417

Wastage	WHO estimates	10%	10%	10%

*Personnel facility level*	[29]	0.06	0.06	0.06

**Waste management**				

**Training over 5 years)**	[29]	**0.03**	**0.03**	**0.03**

**Social mobilization (av)**	[29]	**0.12**	**0.12**	**0.12**

**TOTAL COST PER DOSE**	Derived	**1.63**	**2.86**	**12.66**

*adjusted for inflation

Each simulation is repeated three times using different seeds to initialize the random number generator, and each of these simulations is compared with an independent simulation of a reference scenario: a control population with no vaccine, but with the same human demography, baseline transmission, and health system.

To estimate the number of disability adjusted life years (DALYs), years of life lived with disability are calculated on the basis of the duration of disability, and respective disability weights. Weights for different malaria attributable disease conditions have been obtained from the Global Burden of Disease (GBD) study[25]. DALYs are computed with no age weighting to follow standard cost-effectiveness practices[26]. The disability associated with anemia is assigned to the same time period as the malaria infections causing it.

Years of life lost (YLLs) and DALYs are calculated assuming age-specific life expectancies based on the life-table from Butajira, Ethiopia, with an average life expectancy of 46.6 years at birth[27]. This life-table represents that of an East African setting with low malaria transmission and is very similar to that for Hai District, a high altitude and low malaria prevalence site in Tanzania[28]. YLLs for each simulated death are computed under the assumption that this life table would apply in the absence of malaria.

### Vaccine delivery costs

The vaccine delivery costs are estimated using the methodology of a previous study by the authors[29], which was based on an ingredient approach requiring information on the quantities of physical inputs needed and their unit costs. The costs of introducing a malaria vaccine into the EPI in Tanzania include those related to an assumed range of vaccine purchase costs, and data collected from Tanzania on costs of distribution, cold chain, management, vaccine delivery by health facilities, training, and social mobilization[30] (Table 1). For booster doses, the per-dose delivery cost is assumed to be the same as that of routine EPI.

The costs of vaccine campaigns are estimated by adding to the purchase costs, those costs associated with distribution, cold chain, management, specific programme activities, personnel, and other capital costs, estimated by a study in Tanzania on a campaign for Vitamin A supplementation[31] (Table 2).

**Table 2:** Vaccine delivery costs – Campaign – US$ 2006

**Item**	**Source**	**Costs (US$)**		
Net vacc. purchase cost per dose	Derived	**1.23**	**2.45**	**12.25**

Vaccine price per dose		1	2	10

Freight	UNICEF estimates	0.0417	0.0417	0.0417

Wastage	WHO estimates	15%	15%	15%

Distribution per dose	[29]	**0.09**	**0.09**	**0.09**

Storage per dose	[29]	**0.03**	**0.03**	**0.03**

Management per dose	[29]	**0.003**	**0.003**	**0.003**

Delivery per dose	Derived	**0.07**	**0.07**	**0.07**

*Syringes*		0.06	0.06	0.06

Unit cost per dose	[30]	0.05	0.05	0.05

Freight	UNICEF estimates	0.0417	0.0417	0.0417

Wastage	WHO estimates	10%	10%	10%

*Safety boxes*		0.01	0.01	0.01

Unit cost per dose	[30]	0.0122	0.0122	0.0122

Freight	UNICEF estimates	0.0417	0.0417	0.0417

Wastage	WHO estimates	10%	10%	10%

**Programme-specific costs**		**0.07**	**0.07**	**0.07**

Allowances	[31]	0.1132	0.1132	0.1132
		
Fuel & Maintenance		0.0337	0.0337	0.0337
		
Fax & Telephone		0.0094	0.0094	0.0094
		
Refreshments		0.0058	0.0058	0.0058
		
Stationary & Postage		0.0056	0.0056	0.0056
		
Photocoping		0.0051	0.0051	0.0051
		
Transport		0.0050	0.0050	0.0050
		
Social mobilization		0.0048	0.0048	0.0048
		
Other		0.0005	0.0005	0.0005
		
**Personnel cost**		**0.42**	**0.42**	**0.42**
		
Government		0.3017	0.3017	0.3017
		
Non Government		0.1141	0.1141	0.1141
		
**Capital cost**		**0.07**	**0.07**	**0.07**
		
Vehicles		0.0410	0.0410	0.0410
		
Social mobilization		0.0156	0.0156	0.0156
		
Long term training & studies		0.0153	0.0153	0.0153
		
Other		0.0017	0.0017	0.0017

**TOTAL COST PER DOSE**	Derived	**1.98**	**3.20**	**13.01**

*adjusted for inflation

### Potential cost savings of the interventions

The costs of treating those seeking health care for malaria episodes are calculated under the reference and the vaccine scenarios modeled. This allows calculation of expected cost savings associated with the introduction of an efficacious malaria vaccine. The case management cost inputs correspond to those published previously by the authors adjusted for inflation to 2007[24,32-36] (Table 3) and the first line treatment for uncomplicated malaria changed to an ACT (artemether/lumefantrine), for which the public sector price posted by WHO was used (Table 4).

**Table 3:** Case management unit costs US$ 2006

	**Costs (US$)**	**Source**
**Household average out of pocket costs per outpatient visit**		

Travel costs	0.09	[32]

Medical supplies	0.03	[32]

Non medical supplies	0.22	[32]

Travel costs	0.09	[32]

		

**Unit cost of outpatient visit**	**0.7176**	**derived**

% of outpatient visits that take place at health centers	17%	[33]

% of outpatient visits that take place at dispensaries	72%	[33]

% of outpatient visits that take place at hospitals	10%	[33]

cost per outpatient visits at health centers	1.47	derived

cost per outpatient visits at dispensaries	1.18	derived

cost per outpatient visits at hospitals	2.54	derived

% of patients using Diagnostic Techniques	0.1	[34]

unit cost of Diagnostic Technique	0.3	[34]

% of outpatient visit cost that are recurrent	69%	[32]

% of outpatient visit cost that are fixed	0.25	[32]


		

**Hospital costs of severe episodes**		

Non drug cost per admission when patient fully recorvers	**14.4**	**derived**

Non drug cost per admission when patient recorvers with NS	**32**	**derived**

Non drug cost per admission when patient dies	**6.4**	**derived**

Non drug cost per day of stay	9.00	[36]

Capital	2.60	

Recurrent	6.40	

average length of stay when patient fully recovers	4.5	[41]

average length of stay when patient recovers with NS	10	[41]

average length of stay when patient dies	2	[41]

% of hospital cost that are recurrent	71.1	[35]

% of hospital recurrent costs that are fixed	50.0	[41]

**Table 4:** ACT costs

**Age**	**Cost/dose in $** **(including 12% dist)**	**Cost per course in $** **+ 25% wastage**
<3 years – 5 to 14 Kg	1.008	1.260
3–9 years – 15 to 24 Kg	1.568	1.960
10–14 years 25 to 34 Kg	2.128	2.660
15+ years – Above 35 Kg	2.688	3.360

Source:  accessed 15 July 2008.

The numbers of uncomplicated and severe malaria episodes averted due to vaccination are multiplied by the case management unit costs, as described above, to estimate the potential cost savings for both the health system and households. The cost savings are subtracted from the vaccine costs to compute the net costs of the interventions.

### Calculating cost-effectiveness ratios and interpreting the results

The cost-effectiveness ratios presented are calculated for four health outcomes: uncomplicated and severe malaria episodes averted, DALYs averted, and deaths averted. Future costs and benefits are discounted at 3%. The cost-effectiveness ratios are presented for assumed vaccine prices of US$ 2 or US$ 10 per dose for all the vaccines and vaccine combinations. The results are presented as cost-effectiveness on a 10-year period.

The cost-effectiveness ratios are to be interpreted as incremental cost-effectiveness ratios of implementing the interventions in the simulated scenarios relative to a do-nothing scenario, which corresponds to maintaining only the case management model described above.

### Accounting for uncertainty

The cost-effectiveness results are based on an advanced modeling methodology aimed at representing reality as accurately as possible. The large number of scenarios simulated includes some sensitivity analyses of results for key variables, for instance for vaccine efficacy levels. The likely impact on results of other key features of potential malaria vaccines is explored in the companion article[14].

However, many sources of uncertainty cannot be captured by these sensitivity analyses. Probabilistic sensitivity analysis and expected value of information analysis could serve to further assess the impact of uncertainty on the simulation results [37-40], but there are technical problems in implementing and presenting such analyses for a large set of interventions and scenarios. It has been, therefore, planned to run an expected value of information analysis on a sub-set of simulations that will be reported in another article.

## Results

### Pre-erythrocytic vaccines

**Figure 1:**
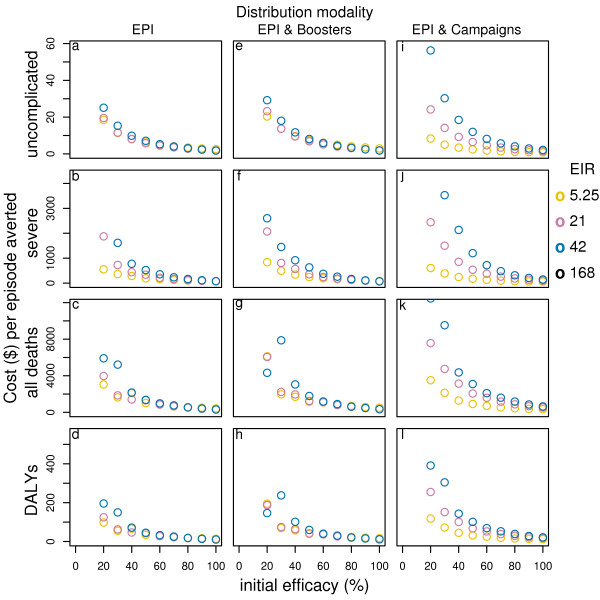
Effect of initial efficacy on cost-effectiveness of PEV by transmission setting and delivery modality*. Results obtained assuming a vaccine half-life of 10 years, homogeneity value of 10, and vaccine price of US$2. EPI & Campaigns means EPI with 70% mass vaccination. *data for EIR in some cases are not shown in the figure due to a scale problem.

At a reference transmission setting with annual entomological inoculation rate (EIR) of 21, the simulations predict that a PEV with 52% initial efficacy could be very cost-effective when delivered via EPI alone. At a vaccine price of US$2 per dose, the cost per uncomplicated malaria episode averted would be around US$ 5, the cost per severe malaria episode averted US$ 269, the cost per DALY averted around US$ 35 and the cost per death averted US$1057 (see table S1 and S2, Additional file 1). The cost-effectiveness ratios are lower for higher effectiveness levels (Figure 1). They increase almost proportionally with vaccine price reaching US$ 160 per DALY averted and US$ 4869 per death averted for a vaccine price of US$ 10 per dose (see table S3 and S4, Additional file 1).

The proportion of events averted by PEV delivered via EPI with booster doses is slightly higher, but the cost per uncomplicated episode averted is 20% higher (see table S1, Additional file 1), and cost per DALY and death averted is around 31% higher (see table S2, Additional file 1).

With EPI and mass vaccination the proportion of events averted is 5% higher for mass vaccination coverage of 50% and 8% higher for coverage of 70%[14], and the cost per uncomplicated episode averted is slightly lower. However, the costs per DALY and death averted are around 60%–66% higher (see table S1 and S2, Additional file 1). For higher efficacy levels the pattern is similar, showing that the incremental benefits of these deployment modalities, in this transmission setting, are modest (Figure 1).

In low transmission settings, while the cost per uncomplicated episode averted under EPI alone is similar to that in the reference transmission setting (see table S1 and S2, Additional file 1), the cost per DALY and death averted are lower at US$ 31 per DALY averted and US$ 925 per death averted at a vaccine price of US$ 2 per dose (see table S2 and S4, Additional file 1). Adding booster doses leads to higher cost-effectiveness ratios for efficacy levels up to around 60%, but at near 100% efficacy the cost-effectiveness ratios become similar (Figure 1). In contrast, when mass vaccination is added to EPI, the cost-effectiveness ratios decrease substantially, by around 70% for the cost per uncomplicated case averted (see table S1 and S3, Additional file 1), and by 24% to 28% for the cost per DALY and death averted (see table S2 and S4, Additional file 1).

In high transmission settings, the effectiveness of PEV is low[14] and the cost-effectiveness ratios are therefore higher than in the other transmission settings irrespective of delivery modality. For some outcomes, vaccination even leads to an increase in the number of clinical events[14], and, therefore, to negative cost-effectiveness ratios and negative case management cost savings (see table S5, Additional file 1).

Across all transmission settings, the incremental benefits of booster doses are small and the cost-effectiveness ratios are higher. Adding mass campaigns has little impact on overall effect when the primary efficacy is low. However, for high vaccine efficacy and high coverage, this strategy is predicted to lead to local elimination of the parasite in low transmission settings and substantially reduce transmission in medium transmission settings[14] at low additional costs. Under these conditions, because of the effects of the vaccine on transmission, delivery via mass campaigns plus EPI becomes a cost-effective alternative to EPI alone.

### Blood-stage vaccines

**Figure 2:**
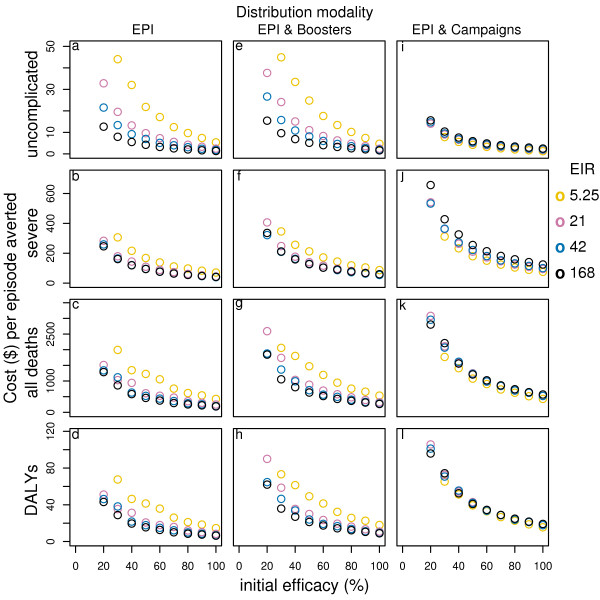
**Effect of initial efficacy on cost-effectiveness of BSV by transmission setting and delivery modality**. Results obtained assuming a vaccine half-life of 10 years, homogeneity value of 10, and vaccine price of US$2. EPI & Campaigns means EPI with 70% mass vaccination.

At the reference transmission intensity, BSV of moderate efficacy with a price of US$ 2 per dose applied through EPI achieves a cost per uncomplicated episode averted of about US$ 9 (see table S1, Additional file 1), which is higher than for the corresponding PEV, but the costs per DALY averted (US$ 21) and per death averted (US$ 630) are lower than for PEV (see table S2, Additional file 1). At higher efficacy levels, the cost-effectiveness ratios decrease, following the same patterns as for PEV (Figure 2). Adding booster doses increases the cost-effectiveness ratios somewhat. Mass campaigns also increase the cost-effectiveness ratios except for uncomplicated episodes, where they decrease.

**Figure 3:**
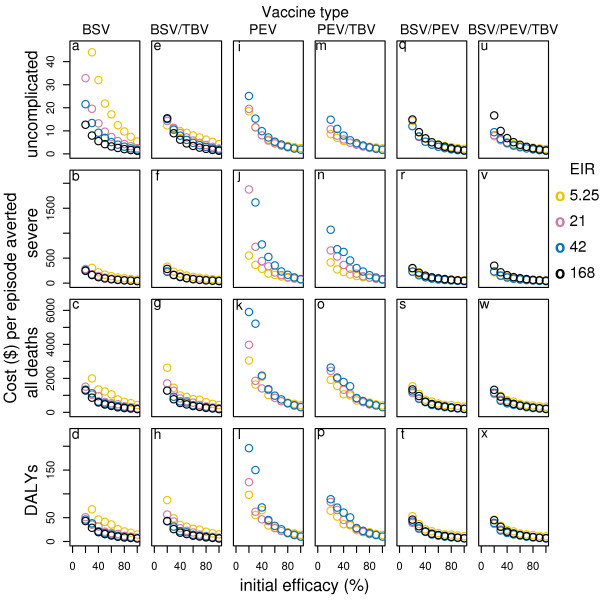
Effect of initial efficacy on cost-effectiveness of all vaccines delivered via EPI by transmission setting*. Results obtained assuming a vaccine half-life of 10 years and homogeneity value of 10, and vaccine price of US$2. *data for EIR in some cases are not shown in the figure due to a scale problem.

**Figure 4:**
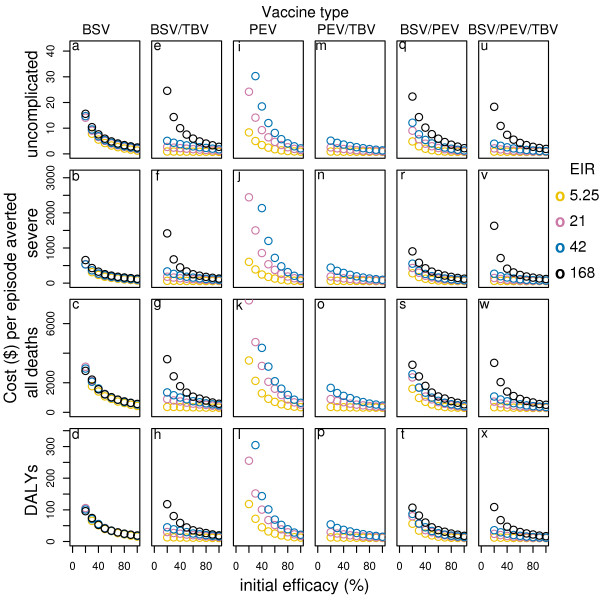
Effect of initial efficacy on cost-effectiveness of all vaccines delivered via EPI with 70-% mass vaccination by transmission setting*. Results obtained assuming a vaccine half-life of 10 years and homogeneity value of 10, and vaccine price of US$2. *data for EIR in some cases are not shown in the figure due to a scale problem.

At low transmission intensity BSV averts a lower proportion of uncomplicated and severe cases and deaths than PEV[14] and the cost effectiveness ratios are higher for all outcomes. Adding booster doses leads to slightly higher costs per uncomplicated episode averted (see table S1 and S3, Additional file 1), and much higher costs per DALY and death averted (see table S2 and S3, Additional file 1, and Figure 2). Adding mass campaigns to EPI leads to a dramatic reduction in the cost per uncomplicated episode averted, but the costs per DALY and death averted are only slightly lower (see table S1, S2, S3, Additional file 1, and Figure 3, 4).

In high transmission settings BSV is more effective than PEV especially in averting severe and mortality events[14] and it is also more efficient. Under EPI alone the cost per uncomplicated episode averted, in the highest transmission setting, is US$ 3.8, the cost per DALY averted is US$13.5 and the cost per death averted is US$401, at vaccine price US$ 2 per dose (see table S1 and S2, Additional file 1, and Figure 3). Adding boosters or mass campaigns, leads to higher incremental costs than incremental benefits (see table S1, S2, S3, Additional file 1, and Figure 2).

Across all transmission settings, the incremental costs of adding booster doses to EPI are higher than the incremental benefits and this is particularly true for severe episodes, DALYs, and mortality (see table S1, S2, S3, Additional file 1, and Figure 2). In low transmission settings, campaigns improve cost-effectiveness for uncomplicated episodes averted, but do not change cost-effectiveness estimates for DALYs and deaths averted. However, in moderate to high transmission settings, the incremental costs of campaigns are higher than the incremental benefits (see table S1, S2, S3, Additional file 1, and Figure 2).

### Combination vaccines and MSTBV

Combining BSV with PEV (with matched efficacies) in general, improves or matches the cases averted over PEV alone for all transmission settings and vaccine delivery modalities[14]. The cost-effectiveness ratios for this combination are lower than those of PEV in all transmission settings particularly for the cost per DALY and per death averted and in moderate to high transmission settings (see table S1, S2, S3 in Additional file 1, and Figure 3, 4). Compared to BSV alone, the cost-effectiveness ratios of combining BSV with PEV are lower, though the difference is smaller than for PEV and in this case it is higher in moderate to lower transmission settings than in high transmission settings. Adding booster doses to EPI leads to higher cost-effectiveness ratios across all transmission settings for this combination – the costs per uncomplicated episode averted increases by around 19%–23% while those per DALY and death averted show even larger increases (around 30%–40%).

Adding mass campaigns in low to moderate settings lead to incremental uncomplicated episodes averted that are higher than the incremental costs. However, in terms of DALYs and deaths averted the benefits exceed the costs only in the lowest transmission setting, while they are significantly lower in the reference and in high transmission settings. In high transmission settings even the additional uncomplicated episodes averted are lower than the additional costs.

Combinations of MS TBV with PEV or BSV and the triple combination do not improve the effectiveness of the vaccines alone when delivered via EPI or EPI with boosters[14]. However, adding mass campaigns leads to greater effectiveness in all transmission settings (Figure 4). The additional benefits of these combination vaccines are then much higher than the additional costs compared to delivering the vaccines under EPI alone and to all delivery modalities of PEV and BSV alone. In the reference transmission setting, for instance, the cost per uncomplicated episode averted of combining BSV with MSTBV, delivered via EPI and mass campaigns, is (at a vaccine price of US$2) US$1.8 and US$2.3 for 70% and 50% coverage (see table S1, Additional file 1), while the cost per DALY averted is US$20 and US$ 22 for 70% and 50% coverage (see table S2, Additional file 1). The costs per DALY averted vary between US$ 12 and US$40 across transmission settings with the lowest value in the lowest transmission setting where the greatest improvement to effectiveness is observed. The very favourable cost-effectiveness ratios in low transmission settings are related to the case-management cost savings, which may compensate up to more than 50% of the costs of the vaccine intervention (see table S4, Additional file 1).

### Effect of delivery modalities

Adding boosters to EPI does not improve effectiveness or cases averted over EPI alone by very much even at the very high coverage level modeled, but it does incur additional costs. This delivery modality does therefore not represent a cost-effective alternative to EPI alone in any scenario (see table S1, S2, S3, Additional file 1).

**Figure 5:**
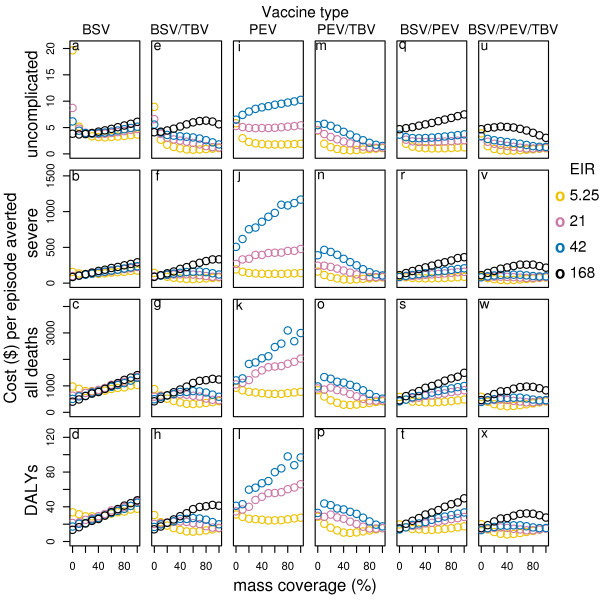
Cost-effectiveness of vaccines given different levels of mass vaccination coverage by transmission setting*. Results obtained assuming a vaccine half-life of 10 years and homogeneity value of 10, and vaccine price of US$2. *data for EIR in some cases are not shown in the figure due to a scale problem.

Delivering all vaccines and combinations via population based campaigns improves the effectiveness at mass vaccination coverage of 50%, especially in low transmission settings[14]. Depending on the transmission setting and the vaccine type considered, the incremental costs of delivering vaccines via population based campaigns can be lower than the incremental benefits, leading to a significant reduction in the cost-effectiveness ratios (see table S1, S2, S3, Additional file 1, and Figure 4). Disseminating vaccines via population-based campaign in these cases is predicted to be a more cost-effective way of delivering malaria vaccines than EPI alone. Increasing the coverage of the mass vaccination campaigns increases the effectiveness and cases averted for all vaccine and vaccine combinations under most transmission settings[14]. However, the incremental benefits of increasing coverage are often lower than the incremental costs of achieving it (Figure 5). In some cases, the predictions suggest an optimal cost-effectiveness ratio at intermediate values for the campaign coverage. This is not a consequence of non-proportionality of vaccine delivery costs as a function of coverage (which could be realistic, but not modeled in this study), but of the indirect effects of the vaccines.

### Effect of vaccine price

Although the simulations focus on comparative cost-effectiveness of different candidate malaria vaccines and delivery modalities, and not on the sensitivity of cost-effectiveness ratios to vaccine prices, which are hypothetical, it is evident that the cost-effectiveness results are almost directly proportional to the vaccine prices. In fact, at an assumed vaccine price of US$ 10 per dose, most cost-effectiveness ratios are between 4 and 7 times higher than those obtained at US$ 2 per dose (see table S1, S2, S3, Additional file 1). At a vaccine price of US$ 2 per dose, most vaccines and delivery modalities simulated present cost-effectiveness ratios comparable to those of other malaria interventions[9,10,41-43], while at a vaccine price of US$ 10 per dose in many of the simulated scenarios the cost-effectiveness ratios are higher.

## Discussion

CEA is a method for evaluating the relative efficiency of alternative interventions and thus can provide important information for assessing the potential implications of the numerous malaria vaccine candidates. This study used stochastic simulations of *P. falciparum *malaria epidemiology, combined with a case management model, to simulate the cost-effectiveness of potential malaria vaccines under various transmission settings and delivered via different modalities. This is an extension of previous research on pre-erythrocytic vaccines delivered via the EPI[12].

The simulations presented suggest that the cost-effectiveness of candidate malaria vaccines is likely to differ substantially according to the transmission intensity and to the delivery modality adopted. They also suggest that alternative vaccine delivery modalities to the EPI may sometimes, but not always, be more cost-effective than the EPI. In general, at moderate vaccine prices, most vaccines and delivery modalities simulated are likely to present cost-effectiveness ratios, which compare favourably with those of other malaria interventions [41-43], making them potential attractive malaria control strategies, from an economic perspective, in malaria endemic countries.

These simulations have various limitations, as described in the companion article on the epidemiological effects[14]. For the economic analysis, one of the most important limitations is related to the relatively simple case management model used to assess the impact of malaria vaccines on the costs to the health system and to patients. As the case management model used is the same for all scenarios simulated, the relative cost-effectiveness of the vaccines modeled, and, therefore, the comparisons, should only be slightly affected by it. However, further research and modeling of health system characteristics in malaria endemic settings is required. Additionally, the vaccine delivery modalities modeled may not be feasible to implement in all settings as the coverage and the effectiveness of malaria vaccines is likely to depend strongly on the characteristics of the health systems where they will be implemented, including any other malaria intervention being delivered. For instance, the simulations assumed an EPI coverage rate of 89%, which is probably higher than found in some malaria-endemic countries. Lower EPI coverage rates could have an impact on the comparisons between different delivery modalities.

Other limitations of this study include that the comparisons of malaria vaccines – or of combinations of them- with different characteristics, are based on the same assumed vaccine price. In practice, the price might vary according to the characteristics of the vaccines, in particular for combinations of vaccines. This might be important for the result that MSTBV combinations were more efficient than vaccines without MSTBV, especially when delivered via EPI with mass campaigns.

While modeling the costs of different vaccine delivery modalities, the fact that vaccine delivery costs might vary as a function of coverage (as it is the case for other interventions[44,45]) was not taken into account. This aspect was not considered due to the lack of solid evidence on vaccine delivery costs by coverage levels, especially for mass campaigns.

Despite these limitations, the simulations presented provide interesting information for vaccine developers on the potentials of different candidate malaria vaccines. Previous simulation of the cost-effectiveness of PEV[12] suggested that at moderate to low vaccine prices, a vaccine providing partial protection, and delivered via the EPI, may be a cost-effective intervention in countries where malaria is endemic. The simulations presented in this article, also show that these types of vaccines are more effective and cost-effective in low transmission settings, and that the additional costs of delivering a PEV under other modalities than the EPI are likely to be higher than the additional health benefits. The only exception is for the scenario of mass vaccination (added to routine EPI) in low transmission and for high vaccine efficacies and high coverage. In contrast to PEV, BSV are predicted to be more effective and cost-effective at higher transmission settings than low transmission.

Combinations of BSV and PEV are predicted to be more efficient than PEV, in particular in moderate to high transmission settings, but compared to BSV, combinations are more cost-effective in mostly moderate to low transmission settings. The cost-effectiveness ratios of the other delivery modalities simulated are higher than those for EPI alone in almost all scenarios, with the exception of adding mass campaigns to EPI in the lowest transmission setting.

Combinations of MSTBV and PEV or PEV and BSV do not increase the effectiveness or the cost-effectiveness compared to PEV and BSV alone when delivered through the EPI (including with the addition of booster doses). However, when applied with EPI and mass vaccinations, combinations with MSTBV provide substantial incremental health benefits and low incremental costs in all transmission settings. These combination vaccines are therefore predicted to be interesting only for the settings where mass vaccination achieving relatively high coverage rates would be feasible.

According to these simulations, adding booster doses to the EPI is unlikely to be a cost-effective alternative to delivering vaccines via the EPI for any vaccine and transmission setting – i.e. the incremental health benefits are rather low despite the additional costs.

Mass vaccination improves effectiveness, especially in low transmission settings, and in some scenario the cost-effectiveness ratios compare favourably with those of delivering the vaccine via the EPI only – the incremental costs are lower than the incremental health benefits. However, increasing the coverage of mass vaccination over 50%, often leads to incremental costs that exceed the incremental health benefits. In some scenarios, the lowest cost-effectiveness ratios are reached at intermediate coverage rates of campaigns. This result is particularly relevant as it is due to the indirect effect of the vaccines, and not to the increasing vaccine delivery costs of achieving high coverage rates.

In some of the mass vaccination scenarios the simulations predict that local elimination of the parasite would be, in principle, possible. In some of these cases, at moderate vaccine prices, the simulations also predict that the cost-effectiveness ratios of achieving local elimination might be relatively low despite the fact that often the incremental costs of achieving high vaccine coverage are higher than the incremental benefits. However, the cost-effectiveness analyses of these simulations include only part of the economic implications of malaria elimination.

If local elimination were feasible, it might be desirable to achieve high vaccine coverage rates even if the incremental costs are high (compared to the incremental health benefits) as elimination would bring future benefits, however to sustain elimination over time, once elimination is achieved there would be a need for strong surveillance and case detection, which would incur substantial additional costs that are not included in our simulations. An assessment of the economic implications of achieving and sustaining local elimination is planned in the next stage of the project.

## Conclusion

The simulations presented supports that cost-effectiveness analyses of candidate malaria vaccines may help guide policy makers and vaccine developers, by providing additional evidence that malaria vaccines may be efficient malaria control interventions. The results also indicate that the transmission setting and the vaccine delivery modality adopted are important determinants of the cost-effectiveness of malaria vaccines. While adding booster doses to the EPI is not a cost-effective alternative to the EPI, mass vaccination is predicted to provide substantial health benefits, in particular in low transmission settings, at low additional costs making such a delivery mode, in principle, attractive and feasible, and in some cases lead to local elimination. Nevertheless, achieving high coverage rates can lead to substantial incremental costs compared to the health benefits, while intermediate coverage rates may be a more efficient use of the resources.

While modeling studies such as this one are useful for exploring the potential impact of malaria vaccines at early stages of development, vaccine development and implementation decisions should be also informed by cost-effectiveness studies carefully tailored to the settings where the vaccines are likely to be adopted.

Ultimately, the relative efficiency of malaria vaccines will depend not only on the characteristics of them but also on the other malaria control interventions implemented. As malaria vaccines will eventually be deployed as part of integrated control strategies, the costs and effects of the interactions of vaccine programmes with those of other malaria control interventions should also be evaluated.

## Competing interests

The authors declare that they have no competing interests.

## Authors' contributions

Conceived and designed the study: FT, MP, NM, TS. Analyzed the data: MP, NM, FT. Wrote the paper: FT, TS. Interpretation of the results: FT, MP, NM, TS. Editing of the manuscript: MP. Programming the simulations: AS, MP, NM. Running the simulations: AS, NM.

## Supplementary Material

Additional file 1 **Cost-effectiveness ratios, cost savings, and net costs**. the data reported in additional file one are the cost-effectiveness ratios, the cost savings and the net costs, of the different vaccines, by delivery modality and transmission settings Click here for file
