# Supplementary material for: Simulation of the cost-effectiveness of malaria vaccines
Source: Malar J. 2009 Jun 8;8:127. doi: 10.1186/1475-2875-8-127 (PMC2701956; doi:10.1186/1475-2875-8-127)
Supplement: Additional file 1 — Cost-effectiveness ratios, cost savings, and net costs. the data reported in additional file one are the cost-effectiveness ratios, the cost savings and the net costs, of the different vaccines, by delivery modality and transmission settings [file 1475-2875-8-127-S1.doc]

***Table S1***

***Cost-effectiveness of different vaccination strategies in US$ per clinical event averted for a range of initial transmission intensities - a vaccine purchase price of 2 US$ per dose is assumed.***

|  |  | Uncomplicated episodes | | | | | Severe episodes | | | | |
| --- | --- | --- | --- | --- | --- | --- | --- | --- | --- | --- | --- |
|  | EIR | EPI | EPI booster | EPI 70% campaign | EPI 50% campaign | EPI 30% campaign | EPI | EPI booster | EPI 70% campaign | EPI 50% campaign | EPI 30% campaign |
| BSV | 5.25 | 19.7 | 22.3 | 3.3 | 3.1 | 3.3 | 156.5 | 200.0 | 152.9 | 136.4 | 128.4 |
|  | 21 | 8.7 | 10.0 | 4.1 | 3.8 | 3.8 | 99.4 | 130.9 | 183.4 | 155.8 | 131.4 |
|  | 84 | 4.6 | 5.6 | 4.7 | 4.2 | 3.9 | 84.5 | 116.0 | 209.6 | 170.8 | 137.8 |
|  | 168 | 3.8 | 4.7 | 5.1 | 4.5 | 4.0 | 85.8 | 117.3 | 226.4 | 187.3 | 147.8 |
| BSV TBV | 5.25 | 8.9 | 10.2 | 0.8 | 0.8 | 1.3 | 143.4 | 176.1 | 60.3 | 56.7 | 75.0 |
|  | 21 | 6.6 | 7.8 | 1.8 | 2.3 | 2.7 | 97.9 | 126.2 | 111.3 | 124.2 | 120.7 |
|  | 84 | 4.6 | 5.6 | 4.3 | 4.3 | 4.1 | 84.1 | 116.5 | 217.7 | 186.1 | 146.8 |
|  | 168 | 4.1 | 5.1 | 6.2 | 5.6 | 4.8 | 87.5 | 122.8 | 284.3 | 224.1 | 162.4 |
| PEV | 5.25 | 5.8 | 6.8 | 1.8 | 1.8 | 2.0 | 191.8 | 228.3 | 129.2 | 128.5 | 135.0 |
|  | 21 | 5.3 | 6.3 | 5.1 | 4.9 | 4.9 | 268.8 | 341.9 | 426.6 | 421.2 | 393.9 |
|  | 84 | 9.9 | 10.8 | 26.9 | 24.7 | 21.0 | neg. | 4'911.5 | neg. | neg. | 8'964.9 |
|  | 168 | 29.2 | 26.7 | neg. | neg. | neg. | neg. | neg. | neg. | neg. | neg. |
| PEV TBV | 5.25 | 4.4 | 5.6 | 0.8 | 0.7 | 0.9 | 162.1 | 206.9 | 59.6 | 49.3 | 65.2 |
|  | 21 | 4.5 | 5.3 | 1.4 | 2.0 | 2.8 | 243.6 | 272.0 | 99.7 | 148.2 | 202.7 |
|  | 84 | 8.4 | 9.2 | 5.0 | 7.6 | 10.4 | 4'436.7 | 2'339.1 | 514.0 | 1'209.2 | 5'559.8 |
|  | 168 | 25.0 | 22.5 | 12.7 | 29.7 | 116.0 | neg. | neg. | neg. | neg. | neg. |
| BSV PEV | 5.25 | 4.7 | 5.8 | 1.1 | 1.0 | 1.2 | 102.4 | 130.8 | 72.5 | 67.1 | 69.0 |
|  | 21 | 3.6 | 4.4 | 2.3 | 2.2 | 2.3 | 82.9 | 106.9 | 136.3 | 122.8 | 106.9 |
|  | 84 | 3.9 | 4.7 | 4.6 | 4.2 | 3.9 | 94.7 | 121.7 | 225.0 | 189.2 | 155.2 |
|  | 168 | 4.7 | 5.6 | 6.5 | 5.9 | 5.4 | 112.1 | 147.3 | 292.3 | 244.5 | 196.4 |
| BSV PEV TBV | 5.25 | 3.9 | 4.9 | 0.8 | 0.6 | 0.7 | 97.1 | 122.3 | 57.2 | 44.4 | 44.4 |
|  | 21 | 3.3 | 4.1 | 1.0 | 1.3 | 1.7 | 81.0 | 102.7 | 72.1 | 83.6 | 90.2 |
|  | 84 | 3.8 | 4.6 | 2.8 | 3.2 | 3.4 | 93.5 | 122.8 | 167.2 | 169.7 | 151.7 |
|  | 168 | 4.7 | 5.5 | 4.4 | 5.0 | 5.2 | 112.2 | 144.9 | 257.9 | 239.4 | 201.5 |

***Table S2***

***Cost-effectiveness of different vaccination strategies in US$ per DALYs and deaths averted for a range of initial transmission intensities - A vaccine purchase price of 2 US$ per dose is assumed.***

|  |  | DALYs | | | | | Deaths | | | | |
| --- | --- | --- | --- | --- | --- | --- | --- | --- | --- | --- | --- |
|  | EIR | EPI | EPI booster | EPI 70% campaign | EPI 50% campaign | EPI 30% campaign | EPI | EPI booster | EPI 70% campaign | EPI 50% campaign | EPI 30% campaign |
| BSV | 5.25 | 33.6 | 47.4 | 33.7 | 29.6 | 29.0 | 976.4 | 1'380.7 | 923.7 | 817.9 | 804.9 |
|  | 21 | 21.4 | 25.6 | 36.8 | 31.8 | 26.7 | 629.7 | 755.6 | 1'073.3 | 921.2 | 783.5 |
|  | 84 | 14.8 | 20.3 | 36.7 | 31.2 | 24.0 | 436.0 | 601.3 | 1'084.1 | 922.4 | 708.3 |
|  | 168 | 13.5 | 19.3 | 37.4 | 29.9 | 23.8 | 400.9 | 572.6 | 1'106.4 | 886.0 | 707.8 |
| BSV TBV | 5.25 | 30.6 | 39.9 | 11.9 | 11.6 | 15.4 | 880.5 | 1'146.9 | 329.2 | 323.0 | 433.9 |
|  | 21 | 20.3 | 26.2 | 20.1 | 22.6 | 22.7 | 591.8 | 770.9 | 593.4 | 666.0 | 669.9 |
|  | 84 | 14.7 | 20.9 | 33.4 | 30.6 | 24.1 | 435.9 | 618.9 | 995.8 | 912.7 | 714.5 |
|  | 168 | 14.1 | 19.3 | 39.8 | 32.6 | 25.5 | 417.6 | 572.1 | 1'187.8 | 970.5 | 757.9 |
| PEV | 5.25 | 31.1 | 41.7 | 24.6 | 24.3 | 25.7 | 925.6 | 1'219.3 | 695.5 | 697.2 | 752.5 |
|  | 21 | 34.8 | 45.9 | 56.9 | 55.7 | 47.9 | 1'057.4 | 1'382.6 | 1'748.6 | 1'707.2 | 1'467.0 |
|  | 84 | 63.7 | 109.7 | 182.3 | 134.7 | 122.8 | 1'936.7 | 3'328.2 | 5'675.0 | 4'173.4 | 3'910.5 |
|  | 168 | 302.6 | 140.8 | 8'658.1 | 325.2 | 438.0 | 15'601.5 | 4'279.4 | neg. | 11'050.7 | 15'568.2 |
| PEV TBV | 5.25 | 28.5 | 37.5 | 11.6 | 9.9 | 12.8 | 834.9 | 1'101.5 | 321.1 | 275.4 | 362.0 |
|  | 21 | 29.7 | 36.3 | 17.1 | 22.9 | 27.8 | 904.2 | 1'084.0 | 508.1 | 691.1 | 840.3 |
|  | 84 | 61.7 | 70.2 | 49.2 | 56.9 | 74.3 | 1'867.9 | 2'095.4 | 1'511.9 | 1'738.7 | 2'280.4 |
|  | 168 | 422.5 | 353.9 | 95.1 | 163.1 | 277.3 | 20'445.9 | 19'392.0 | 2'967.8 | 5'400.1 | 9'418.8 |
| BSV PEV | 5.25 | 19.7 | 27.6 | 14.5 | 13.4 | 14.2 | 581.3 | 825.4 | 402.1 | 373.4 | 400.1 |
|  | 21 | 15.1 | 19.5 | 24.3 | 21.5 | 18.8 | 445.6 | 579.0 | 717.3 | 638.0 | 555.0 |
|  | 84 | 15.3 | 19.9 | 33.7 | 28.7 | 23.9 | 455.9 | 591.2 | 1'004.5 | 860.3 | 712.0 |
|  | 168 | 15.2 | 20.7 | 40.1 | 33.4 | 27.1 | 453.1 | 615.5 | 1'196.4 | 998.7 | 804.9 |
| BSV PEV TBV | 5.25 | 20.0 | 25.3 | 11.0 | 8.7 | 8.9 | 586.5 | 742.4 | 303.3 | 241.4 | 249.8 |
|  | 21 | 13.8 | 18.1 | 12.9 | 14.7 | 16.2 | 409.2 | 535.6 | 379.1 | 433.0 | 482.5 |
|  | 84 | 14.6 | 18.6 | 24.4 | 24.0 | 22.4 | 434.5 | 552.5 | 731.7 | 717.7 | 668.5 |
|  | 168 | 15.5 | 20.4 | 32.4 | 29.8 | 26.5 | 463.1 | 606.1 | 974.9 | 892.4 | 792.7 |

***Table S3***

***Cost-effectiveness of different vaccination strategies in US$ per clinical event averted for a range of initial transmission intensities - a vaccine purchase price of 10 US$ per dose is assumed*.**

|  |  | Uncomplicated | | | | | Severe episodes | | | | |
| --- | --- | --- | --- | --- | --- | --- | --- | --- | --- | --- | --- |
|  | EIR | EPI | EPI booster | EPI 70% campaign | EPI 50% campaign | EPI 30% campaign | EPI | EPI booster | EPI 70% campaign | EPI 50% campaign | EPI 30% campaign |
| BSV | 5.25 | 91.6 | 102.8 | 15.2 | 14.7 | 15.7 | 729.5 | 923.2 | 702.6 | 635.6 | 605.7 |
|  | 21 | 41.8 | 47.4 | 18.5 | 17.5 | 17.6 | 477.1 | 618.2 | 821.3 | 709.1 | 609.8 |
|  | 84 | 22.6 | 27.0 | 20.9 | 19.1 | 17.9 | 414.7 | 555.0 | 926.3 | 767.7 | 633.6 |
|  | 168 | 18.8 | 22.7 | 22.3 | 19.9 | 18.1 | 421.0 | 562.1 | 995.5 | 836.3 | 675.9 |
| BSV TBV | 5.25 | 42.2 | 47.7 | 4.6 | 4.7 | 6.8 | 677.5 | 823.4 | 330.1 | 313.2 | 386.1 |
|  | 21 | 31.8 | 37.0 | 8.4 | 10.8 | 12.8 | 471.7 | 599.0 | 522.5 | 576.0 | 564.2 |
|  | 84 | 22.7 | 26.9 | 19.0 | 19.3 | 18.7 | 412.2 | 556.6 | 954.2 | 828.2 | 668.4 |
|  | 168 | 20.1 | 24.4 | 27.0 | 24.7 | 21.6 | 427.6 | 585.4 | 1'230.0 | 986.0 | 733.2 |
| PEV | 5.25 | 27.2 | 31.7 | 8.5 | 8.5 | 9.6 | 897.2 | 1'061.4 | 613.7 | 613.9 | 643.0 |
|  | 21 | 24.4 | 28.9 | 21.7 | 21.2 | 21.4 | 1'238.0 | 1'564.3 | 1'835.1 | 1'824.9 | 1'725.9 |
|  | 84 | 43.9 | 47.8 | 111.8 | 103.3 | 88.9 | neg. | 21'797.5 | neg. | neg. | 37'938.5 |
|  | 168 | 126.8 | 117.0 | neg. | neg. | neg. | neg. | neg. | neg. | neg. | neg. |
| PEV TBV | 5.25 | 21.0 | 26.4 | 4.5 | 4.0 | 5.0 | 768.7 | 967.4 | 328.0 | 285.0 | 351.8 |
|  | 21 | 20.9 | 24.6 | 6.7 | 9.2 | 12.5 | 1'129.4 | 1'253.8 | 479.0 | 680.6 | 914.0 |
|  | 84 | 37.4 | 41.0 | 21.5 | 31.9 | 43.9 | 19'645.3 | 10'396.4 | 2'186.7 | 5'107.2 | 23'544.5 |
|  | 168 | 108.3 | 98.1 | 52.8 | 122.7 | 483.9 | neg. | neg. | neg. | neg. | neg. |
| BSV PEV | 5.25 | 22.7 | 27.4 | 5.6 | 5.5 | 6.2 | 495.9 | 623.7 | 378.6 | 355.1 | 361.4 |
|  | 21 | 17.8 | 21.3 | 10.8 | 10.4 | 10.7 | 406.5 | 513.2 | 624.3 | 569.9 | 504.3 |
|  | 84 | 18.9 | 22.4 | 20.1 | 18.7 | 17.7 | 456.8 | 577.5 | 983.9 | 838.5 | 701.3 |
|  | 168 | 22.3 | 26.1 | 28.1 | 25.7 | 23.8 | 532.4 | 689.9 | 1'261.7 | 1'068.4 | 873.9 |
| BSV PEV TBV | 5.25 | 19.1 | 23.7 | 4.4 | 3.7 | 4.0 | 475.2 | 586.7 | 318.4 | 265.2 | 262.9 |
|  | 21 | 16.2 | 19.6 | 5.2 | 6.6 | 8.2 | 398.4 | 495.3 | 365.8 | 409.3 | 435.4 |
|  | 84 | 18.2 | 21.7 | 12.3 | 14.2 | 15.6 | 450.7 | 581.8 | 745.0 | 756.6 | 685.9 |
|  | 168 | 22.2 | 25.9 | 19.2 | 21.9 | 23.0 | 532.1 | 678.8 | 1'117.7 | 1'044.5 | 892.5 |

***Table S4***

***Cost-effectiveness of different vaccination strategies in US$ per DALYs and deaths averted for a range of initial transmission intensities - a vaccine purchase price of 10 US$ per dose is assumed.***

|  |  | DALYs | | | | | Deaths | | | | |
| --- | --- | --- | --- | --- | --- | --- | --- | --- | --- | --- | --- |
|  | EIR | EPI | EPI booster | EPI 70% campaign | EPI 50% campaign | EPI 30% campaign | EPI | EPI booster | EPI 70% campaign | EPI 50% campaign | EPI 30% campaign |
| BSV | 5.25 | 156.7 | 218.8 | 154.9 | 138.1 | 136.6 | 4'550.1 | 6'371.6 | 4'244.1 | 3'811.6 | 3'797.5 |
|  | 21 | 102.5 | 121.1 | 164.8 | 144.5 | 123.7 | 3'022.8 | 3'567.7 | 4'807.3 | 4'191.1 | 3'635.6 |
|  | 84 | 72.5 | 97.2 | 162.1 | 140.1 | 110.2 | 2'140.5 | 2'877.6 | 4'792.2 | 4'146.7 | 3'257.2 |
|  | 168 | 66.4 | 92.4 | 164.3 | 133.4 | 108.7 | 1'968.1 | 2'745.1 | 4'864.2 | 3'956.9 | 3'236.2 |
| BSV TBV | 5.25 | 144.3 | 186.3 | 65.2 | 64.3 | 79.5 | 4'158.8 | 5'361.1 | 1'801.3 | 1'782.5 | 2'233.9 |
|  | 21 | 97.6 | 124.6 | 94.4 | 104.6 | 106.3 | 2'851.7 | 3'660.0 | 2'786.3 | 3'087.3 | 3'131.1 |
|  | 84 | 72.2 | 99.9 | 146.3 | 136.3 | 109.9 | 2'136.7 | 2'958.4 | 4'365.6 | 4'061.3 | 3'253.3 |
|  | 168 | 68.9 | 91.8 | 172.3 | 143.6 | 115.1 | 2'042.0 | 2'727.9 | 5'139.1 | 4'271.0 | 3'421.6 |
| PEV | 5.25 | 145.7 | 193.7 | 116.8 | 116.0 | 122.6 | 4'328.7 | 5'669.8 | 3'304.2 | 3'329.5 | 3'583.2 |
|  | 21 | 160.2 | 210.2 | 244.7 | 241.2 | 209.9 | 4'869.9 | 6'327.3 | 7'522.9 | 7'396.8 | 6'429.6 |
|  | 84 | 281.9 | 486.8 | 757.8 | 563.1 | 518.3 | 8'564.8 | 14'776.1 | 23'590.3 | 17'448.8 | 16'506.1 |
|  | 168 | 1'314.1 | 616.9 | 35'637.1 | 1'344.9 | 1'827.5 | 67'815.3 | 18'744.6 | neg. | 45'698.5 | 64'956.9 |
| PEV TBV | 5.25 | 135.3 | 175.6 | 63.9 | 57.4 | 69.0 | 3'958.6 | 5'151.2 | 1'768.3 | 1'593.7 | 1'952.6 |
|  | 21 | 137.8 | 167.3 | 82.2 | 105.3 | 125.3 | 4'193.4 | 4'996.4 | 2'441.7 | 3'174.4 | 3'788.9 |
|  | 84 | 273.2 | 312.4 | 209.2 | 240.2 | 315.2 | 8'274.7 | 9'330.0 | 6'433.3 | 7'345.5 | 9'671.0 |
|  | 168 | 1'829.4 | 1'545.4 | 394.7 | 674.7 | 1'155.2 | 88'498.2 | 84'676.3 | 12'317.4 | 22'343.9 | 39'230.8 |
| BSV PEV | 5.25 | 95.5 | 131.4 | 75.8 | 71.0 | 74.3 | 2'813.1 | 3'934.7 | 2'098.9 | 1'974.8 | 2'095.1 |
|  | 21 | 74.2 | 93.8 | 111.2 | 100.0 | 88.6 | 2'185.5 | 2'780.8 | 3'285.9 | 2'961.3 | 2'618.3 |
|  | 84 | 74.0 | 94.5 | 147.2 | 127.3 | 108.0 | 2'200.0 | 2'806.4 | 4'393.0 | 3'812.9 | 3'216.3 |
|  | 168 | 72.1 | 97.1 | 173.1 | 146.2 | 120.4 | 2'151.6 | 2'883.4 | 5'164.4 | 4'364.3 | 3'581.7 |
| BSV PEV TBV | 5.25 | 97.9 | 121.2 | 61.1 | 52.2 | 52.9 | 2'869.6 | 3'560.7 | 1'689.1 | 1'442.9 | 1'477.8 |
|  | 21 | 68.0 | 87.4 | 65.6 | 71.7 | 78.2 | 2'013.4 | 2'583.7 | 1'922.0 | 2'119.2 | 2'329.1 |
|  | 84 | 70.2 | 88.2 | 108.9 | 106.9 | 101.1 | 2'094.7 | 2'617.3 | 3'260.0 | 3'199.3 | 3'022.8 |
|  | 168 | 73.7 | 95.5 | 140.3 | 130.0 | 117.5 | 2'196.0 | 2'838.8 | 4'225.6 | 3'894.2 | 3'511.0 |

***Table S5***

***Net cost and cost savings of different vaccination strategies - A vaccine purchase price of 2 US$ per dose is assumed –values discounted at 3%***

|  |  | Net cost | | | | | Cost savings | | | | |
| --- | --- | --- | --- | --- | --- | --- | --- | --- | --- | --- | --- |
|  | EIR | EPI | EPI booster | EPI 70% campaign | EPI 50% campaign | EPI 30% campaign | EPI | EPI booster | EPI 70% campaign | EPI 50% campaign | EPI 30% campaign |
| BSV | 5.25 | 392.9 | 554.8 | 1'119.2 | 898.4 | 685.5 | 27.0 | 30.7 | 144.5 | 125.3 | 96.7 |
|  | 21 | 378.3 | 539.6 | 1'156.0 | 925.0 | 699.7 | 41.3 | 46.5 | 107.3 | 97.2 | 82.0 |
|  | 84 | 367.6 | 530.3 | 1'175.9 | 939.8 | 707.8 | 51.8 | 55.6 | 87.4 | 82.8 | 74.0 |
|  | 168 | 368.0 | 529.5 | 1'184.2 | 947.1 | 712.2 | 51.8 | 56.7 | 79.1 | 74.8 | 68.6 |
| BSV TBV | 5.25 | 386.5 | 545.6 | 899.6 | 726.4 | 613.8 | 33.6 | 39.6 | 363.8 | 295.4 | 167.8 |
|  | 21 | 376.7 | 535.2 | 1'088.4 | 902.9 | 692.8 | 43.1 | 50.3 | 174.9 | 119.1 | 88.4 |
|  | 84 | 368.7 | 531.2 | 1'188.6 | 951.0 | 716.4 | 51.1 | 54.8 | 74.8 | 70.3 | 64.8 |
|  | 168 | 369.6 | 532.5 | 1'209.0 | 964.9 | 724.5 | 50.0 | 53.1 | 54.3 | 56.6 | 57.0 |
| PEV | 5.25 | 390.6 | 550.1 | 1'072.3 | 870.3 | 677.3 | 28.8 | 35.9 | 191.0 | 152.7 | 104.7 |
|  | 21 | 398.4 | 561.3 | 1'217.4 | 984.7 | 752.9 | 21.0 | 24.6 | 45.3 | 37.0 | 28.8 |
|  | 84 | 420.3 | 583.5 | 1'274.3 | 1'033.0 | 790.7 | (0.5) | 2.2 | (11.0) | (10.3) | (8.5) |
|  | 168 | 431.3 | 594.1 | 1'289.8 | 1'046.6 | 803.0 | (11.6) | (8.4) | (27.3) | (25.5) | (20.9) |
| PEV TBV | 5.25 | 384.0 | 546.1 | 892.6 | 686.0 | 579.4 | 35.4 | 39.9 | 370.9 | 336.2 | 202.1 |
|  | 21 | 395.3 | 556.0 | 1'056.4 | 914.5 | 724.9 | 24.5 | 29.7 | 206.3 | 108.5 | 55.7 |
|  | 84 | 419.5 | 581.1 | 1'235.0 | 1'017.9 | 785.8 | 0.3 | 4.4 | 27.7 | 3.9 | (4.1) |
|  | 168 | 430.8 | 596.3 | 1'276.4 | 1'046.4 | 804.0 | (11.1) | (10.4) | (13.4) | (24.1) | (21.6) |
| BSV PEV | 5.25 | 374.8 | 532.6 | 952.3 | 765.7 | 601.2 | 45.5 | 53.1 | 310.1 | 256.6 | 180.6 |
|  | 21 | 368.5 | 528.1 | 1'122.8 | 901.7 | 684.2 | 51.4 | 57.9 | 140.2 | 120.5 | 96.4 |
|  | 84 | 376.1 | 535.8 | 1'192.4 | 956.4 | 723.2 | 43.9 | 50.1 | 71.0 | 65.4 | 57.5 |
|  | 168 | 383.4 | 545.2 | 1'212.5 | 974.1 | 738.7 | 36.0 | 41.0 | 50.6 | 47.8 | 43.3 |
| BSV PEV TBV | 5.25 | 369.8 | 529.2 | 880.8 | 659.8 | 518.0 | 50.3 | 57.1 | 383.2 | 362.8 | 263.7 |
|  | 21 | 366.8 | 525.4 | 987.6 | 842.2 | 664.6 | 52.9 | 61.0 | 275.1 | 178.8 | 115.9 |
|  | 84 | 376.3 | 536.7 | 1'163.1 | 949.3 | 722.0 | 43.4 | 48.7 | 99.4 | 72.5 | 58.3 |
|  | 168 | 384.4 | 545.4 | 1'206.1 | 977.1 | 742.8 | 35.4 | 41.0 | 57.1 | 46.1 | 39.1 |

The total cost is the net cost of the intervention, considering the cost savings due to the aversion of clinical episodes as shown in the right-hand side of the table. Negative numbers indicate higher costs (for case management) in the intervention scenario. The numbers are given per 1000 simulated person-years.
